# Bio-nanoparticles loaded with synovial-derived exosomes ameliorate osteoarthritis progression by modifying the oxidative microenvironment

**DOI:** 10.1186/s12951-024-02538-w

**Published:** 2024-05-20

**Authors:** Haifei Cao, Wanxin Li, Hao Zhang, Lihui Hong, Xiaoxiao Feng, Xuzhu Gao, Hongye Li, Nanning Lv, Mingming Liu

**Affiliations:** 1 Department of Orthopaedics, Yantai Affiliated Hospital of Binzhou Medical University, Yantai, 264000 China; 2 Department of Orthopedic Surgery, The Second People’s Hospital of Lianyungang, The Affiliated Lianyungang Clinical College of Xuzhou Medical University, Lianyungang, 222003 China; 3 Department of Orthopedic Surgery, The Affiliated Lianyungang Clinical College of Jiangsu University, Lianyungang, 222003 China; 4 Department of Otolaryngology Head and Neck Surgery, Beijing Friendship Hospital, Capital Medical University, 95th Yong’an Road, Xicheng District, Beijing, 100050 China

**Keywords:** Osteoarthritis, Exosomes, SOD3, Microspheres

## Abstract

**Background and aims:**

Osteoarthritis (OA) is a prevalent degenerative joint disorder, marked by the progressive degeneration of joint cartilage, synovial inflammation, and subchondral bone hyperplasia. The synovial tissue plays a pivotal role in cartilage regulation. Exosomes (EXOs), small membrane-bound vesicles released by cells into the extracellular space, are crucial in mediating intercellular communication and facilitating the exchange of information between tissues. Our study aimed to devise a hydrogel microsphere infused with SOD3-enriched exosomes (S-EXOs) to protect cartilage and introduce a novel, effective approach for OA treatment.

**Materials and methods:**

We analyzed single-cell sequencing data from 4247 cells obtained from the GEO database. Techniques such as PCR, Western Blot, immunofluorescence (IF), and assays to measure oxidative stress levels were employed to validate the cartilage-protective properties of the identified key protein, SOD3. In vivo, OA mice received intra-articular injections of S-EXOs bearing hydrogel microspheres, and the effectiveness was assessed using safranine O (S.O) staining and IF.

**Results:**

Single-cell sequencing data analysis suggested that the synovium influences cartilage via the exocrine release of SOD3. Our findings revealed that purified S-EXOs enhanced antioxidant capacity of chondrocytes, and maintained extracellular matrix metabolism stability. The S-EXO group showed a significant reduction in mitoROS and ROS levels by 164.2% (*P* < 0.0001) and 142.7% (*P* < 0.0001), respectively, compared to the IL-1β group. Furthermore, the S-EXO group exhibited increased COL II and ACAN levels, with increments of 2.1-fold (*P* < 0.0001) and 3.1-fold (*P* < 0.0001), respectively, over the IL-1β group. Additionally, the S-EXO group showed a decrease in MMP13 and ADAMTS5 protein expression by 42.3% (*P* < 0.0001) and 44.4% (*P* < 0.0001), respectively. It was found that S-EXO-containing hydrogel microspheres could effectively deliver SOD3 to cartilage and significantly mitigate OA progression. The OARSI score in the S-EXO microsphere group markedly decreased (*P* < 0.0001) compared to the OA group.

**Conclusion:**

The study demonstrated that the S-EXOs secreted by synovial fibroblasts exert a protective effect on chondrocytes, and microspheres laden with S-EXOs offer a promising therapeutic alternative for OA treatment.

**Supplementary Information:**

The online version contains supplementary material available at 10.1186/s12951-024-02538-w.

## Introduction

Osteoarthritis (OA) is a chronic degenerative condition distinguished by the progressive degradation of articular cartilage within joints, resulting in pain and functional limitations [1]. The onset and progression of OA involve complex interactions among the cartilage, synovial membrane, and subchondral bone [2]. Cartilage depletion exposes the underlying bone to increased stress, causing micro-damage and subsequent subchondral bone remodeling [3]. This remodeling process includes changes in bone density, increased bone stiffness, and the formation of cysts or bone marrow lesions, significantly contributing to joint pain and dysfunction [4, 5]. Additionally, the synovial membrane plays a crucial role in OA’s inflammatory response, with the inflamed synovium releasing pro-inflammatory cytokines and enzymes that further degrade the cartilage and promote joint inflammation [6–8]. This creates a vicious cycle of cartilage degradation, synovial inflammation, and subchondral bone changes, intensifying OA symptoms [9]. The limited vascularization and lymphatic presence in cartilage tissue impede its regenerative capacity, further challenging the development of effective strategies in clinical practice [10].

Oxidative stress is a crucial factor in the advancement of OA [11]. In OA, oxidative stress [12] is known to cause damage to cartilage by directly degrading its extracellular matrix components, including collagen and proteoglycans, which are essential for maintaining structural integrity and functionality [13]. This leads to decreased cartilage thickness and elasticity, resulting in joint pain and stiffness. Furthermore, oxidative stress can disrupt the balance between anabolic and catabolic processes in the joint [14], impairing the synthesis of matrix components and reducing the effectiveness of anabolic factors, while enhancing the expression of catabolic factors [15]. This imbalance accelerates cartilage degradation and hinders its repair mechanisms [16]. Given these insights, investigating interventions that target oxidative stress and its related pathways could offer a promising direction for developing new therapeutic strategies. Synovial tissue, critical for regulating cartilage health and function in both normal and inflammatory states [17], lines the inner surface of joint capsules and produces synovial fluid. This fluid not only lubricates the joint but also delivers essential nutrients and growth factors that support and maintain articular cartilage [18]. Despite its key role, the extent to which synovial tissue can enhance chondrocytes’ antioxidant capacity and exert a chondroprotective effect remains to be fully elucidated.

Exosomes (EXOs) are diminutive vesicles enclosed by membranes that are secreted by cells into the extracellular milieu, serving as pivotal mediators of intercellular communication and the transmission of molecular signals among diverse tissues [19]. These vesicles are secreted by various cell types, including immune, stem, and cancer cells, and they carry a diverse array of contents such as proteins, nucleic acids (DNA, mRNA, and microRNAs), and other biologically active compounds. EXOs are integral to the complex interactions among different tissues, significantly impacting tissue repair and regeneration [20–22]. Additionally, in the context of tissue injury or inflammation, cells release EXOs loaded with anti-inflammatory agents, thereby helping to modulate the inflammatory response and support tissue homeostasis [23].

Hydrogel microspheres, crafted using microfluidic technology, enable precise control and manipulation of hydrogels at the micron scale. These microspheres are an emerging therapeutic approach for arthritis management, offering targeted and sustained drug delivery directly to the affected joints [24]. Their controlled release mechanism ensures a stable drug concentration within the joint, reducing the frequency of injections and maintaining consistent therapeutic effects [25]. Moreover, microspheres serve as carriers for various therapeutic agents, including growth factors, cytokines, and gene therapy vectors, enhancing tissue repair, modulating immune responses, and regulating inflammatory processes in the joint [26]. This comprehensive strategy holds considerable promise for arthritis treatment, allowing for the integration of multiple therapeutic interventions.

**Scheme 1:**
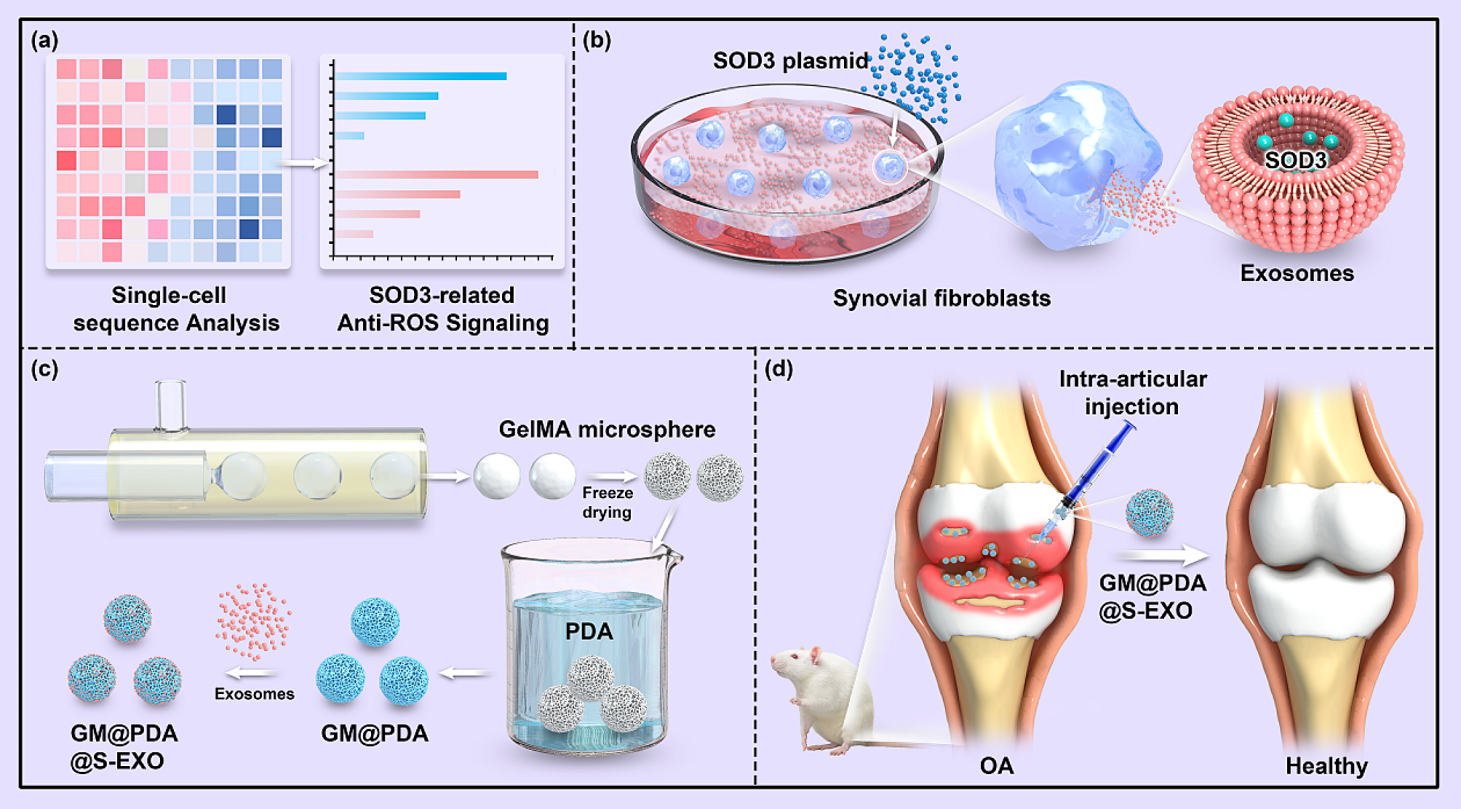
The process of our study. (**a**) Analysis of single-cell sequencing data. (**b**) Preparation of S-EXOs. (**c**) Preparation of hydrogel microspheres loaded with S-EXOs. (**d**) Intra-articular injection of hydrogel microspheres loaded with S-EXOs for OA treatment

The aim of our research was to employ single-cell sequencing technology to identify a potential key regulatory factor of synovial fibroblasts on chondrocytes and to evaluate its influence on chondrocyte antioxidant activity and cartilage matrix metabolism in vitro. Subsequently, hydrogel microspheres loaded with superoxide dismutase 3 (SOD3)-enriched EXOs (S-EXOs) were intra-articular injected to OA mice for assessing their therapeutic efficacy on cartilage (Scheme 1).

## Materials and methods

### **Analysis of single-cell RNA-sequence data**

The single-cell transcriptome analysis utilized original data sourced from the Gene Expression Omnibus (GEO) database (https://www.ncbi.nlm.nih.gov/geo/) (Dataset number: GSE176308). The Seurat software package facilitated object generation and the exclusion of low-quality cells. Subsequent steps involved standard data preprocessing algorithms, which included calculating gene count, cell count, and mitochondrial sequencing count percentages. Cells with a gene count ranging from 200 to 6,000 were considered. To normalize library size effects across cells, unique molecular identifier counts were scaled using a factor of 10,000. The processed normalized data were then used for subsequent analyses as prescribed by the Seurat package. Principal component analysis was conducted on the top 20 variable genes, retaining the first six principal components for further uniform manifold approximation and projection (UMAP) visualization and clustering.

### Cell culture

Mouse synovial fibroblasts, sourced from Hycyte (PCC-C224, Suzhou, China), were treated with 100 nM SOD3 mimics (GenePharma, Shanghai, China) or with 100 nM control substances. The sequences for SOD3 mimics and the negative control are documented in Supplementary Table 1. Chondrocytes were harvested from the cartilage of 8-week-old mice. The tissue was excised meticulously using a sterile blade and then the cartilage was minced into 1-mm^3 fragments under a microscope. The fragments were subsequently subjected to digestion in DMEM/F12 medium supplemented with 2 mg/mL type II collagenase (Thermo) for a duration of 6 h. The chondrocytes were digested, centrifuged, and then cultured in DMEM/F12 medium with 10% FBS. For subsequent experiments, chondrocytes at passage two were treated with IL-1β (10 ng/mL) and exosomes (EXOs or S-EXOs, 10 µg/mL).

### Isolation of exosomes

Exosomes were isolated from mouse synovial fibroblasts at passages 3–6, extracted from the supernatant of 2 × 10^7^ cell cultures. The medium was initially centrifuged at 300 g for 10 min to remove cells, then at 3,000 g for 15 min to discard cell debris. The supernatant was further centrifuged at 120,000 g for 1 h. The pelleted particles were washed in PBS to remove contaminants, followed by a final centrifugation at 120,000 g for 70 min. The purified particles were resuspended in 200 µL of PBS and stored at -80 °C. Transmission electron microscopy (TEM, Leica, Germany) assessed the morphology and size distribution, while nanoparticle tracking analysis (NTA, Malvern, Worcestershire, UK) measured the particle size. Western blotting was performed to detect exosomal markers CD9, CD63, and TSG101, and Calnexin in the cell lysate.

### qRT-PCR

Trizol reagent (Beyotime) was applied to extract total RNA. The synthesis of complementary DNA (cDNA) was conducted utilizing a cDNA synthesis kit from Beyotime, with the reaction being initiated using the SYBR Green kit from Bio-Rad, CA. The relative levels of target genes (*Sod2*, *Sod3*, *Col2a1*, *Acan*, *Mmp13*, *Adamts5*) were quantified using Gapdh as the reference, employing the formula χ = 2^-ΔΔCT^. Primer sequences used in our study were recorded in Supplemental Table 2.

### Western blot

Proteins were isolated using RIPA lysis buffer with protease inhibitors (Beyotime). The concentration of the protein samples was tested using the BCA kit (Beyotime). An equivalent quantity of protein underwent electrophoresis on a 10% SDS-PAGE gel and subsequently underwent transfer to a nitrocellulose membrane. After blocking at room temperature for 1 h, the membranes were incubated with primary antibodies overnight at 4 °C. The antibodies used were COL II (Abcam, ab188570), ACAN (Abcam, ab315486), MMP13 (Abcam, ab39012), ADAMTS5 (Abcam, ab41037), β-actin (Abcam, ab8226), CD9 (CST, 98,327 S), CD63 (Immunoway, YT5525), SOD3 (Santa, 271,170), Calnexin (Affinity, AF5362), and TSG101 (Abcam, ab125011). The subsequent day, the membranes were incubated with HRP-conjugated secondary antibodies (Abcam, ab6721 or ab6788) at room temperature for a duration of 1 h. Detection was achieved using a highly sensitive chemiluminescence substrate (Beyotime), and optical density (OD) values were quantitatively analyzed using β-actin as the loading control.

### Immunofluorescence staining

Chondrocytes were fixed in 4% polyformaldehyde solution (Aladdin, Shanghai, China) for 30 min and permeabilized with Triton X-100 solution (Beyotime) for 10 min. After a 30-minute blocking, the cells were incubated with primary antibodies at 4 °C overnight. After incubating with secondary antibodies (Abcam, ab150077 or ab150079), cell nuclei were stained with DAPI solution (Beyotime) and examined under a fluorescence microscope (Zeiss).

### Detection of antioxidant capacity

Chondrocytes were divided into four groups: CTRL, IL-1β, IL-1β + EXOs, and IL-1β + S-EXOs. The IL-1β group was stimulated with 10 ng/mL of IL-1β, while the IL-1β + EXOs and IL-1β + S-EXOs groups received IL-1β and exosomes (EXOs and S-EXOs, 10 µg/mL) treatment. Intracellular ROS levels were assessed by treating cells with 5 µM DCFH-DA (Beyotime) for 20 min at 37 °C. Mitochondrial ROS were evaluated using MitoSOX solution (5 µM, Beyotime). Fluorescence microscopy (Zeiss) was used to visualize the cells. In the DPPH assay, a mixture of 2 mL cell medium and 2 mL DPPH solution (0.04 mg/mL) was incubated for 30 min, followed by absorbance measurement at 515 nm using UV-visible spectroscopy. For the ABTS assay, a 0.2 mL ABTS (7.4 mM) solution was mixed with 0.2 mL potassium persulfate solution (2.6 mmol/L), incubated in the dark for 24 h to form ABTS+, then diluted 50-fold with PBS and mixed with the medium for a 10-minute reaction before measuring absorbance at 734 nm. In the PTIO experiment, 2 mL of PTIO solution (0.05 mg/mL) was added to each group’s medium and incubated at room temperature for 2 h, with absorbance measured at 557 nm. The hydroxyl radical scavenging assay was conducted following the kit’s instructions (Solarbio, Beijing, China), and absorbance at 550 nm was measured to quantify hydroxyl radical levels.

### Preparation and characterization of EXOs-loaded microspheres

A mixture containing 10 wt% GelMA, a photoinitiator, and 5 wt% Span 80 oil was processed in a microfluidic device to create uniformly sized droplets. The crosslinking of these droplets was achieved through UV irradiation. The resultant microspheres were washed with 75% ethanol solution and stored in deionized water. For surface modification, GelMA microspheres (GMs) were soaked in a polydopamine (PDA) solution (2 mg/mL in 10 mM Tris HCl, pH 8.5) for 6 h, forming GM@PDA. The size and morphology of the microspheres were examined using an optical microscope, and the structure of the freeze-dried microspheres was analyzed with a scanning electron microscope (SEM). Elemental mapping was conducted with an exposure time of 180 s.

### Exosome adsorption and release assay

The exosome adsorption process was performed in sterile conditions. Five milliliters of extracellular vesicles (1,000 µg/mL) were mixed with 100 mg of microspheres and incubated at 4^o^C. The adsorption kinetics of the extracellular vesicles were monitored over specified intervals. At each time point, the supernatant was collected for analysis. To evaluate the release rate of extracellular vesicles, a complex comprising 5 mg of microspheres and extracellular vesicles was submerged in 100 µL of PBS and incubated on a horizontal shaker at 37^o^C and 1,000 rpm. The supernatant was collected at designated time points and refreshed with new PBS. The concentration of EXOs in the supernatant was determined through the BCA kit.

### Cells proliferation and viability

Cell proliferation was evaluated using the Cell Count Kit-8 (CCK-8, Beyotime, Haimen, China). Chondrocytes were incubated with various microspheres (GM@PDA, GM@PDA@EXO, or GM@PDA@S-EXO, 2 mg/well) and the CCK-8 solution at 37 °C. After 1 h, the OD was measured with a multi-plate reader (BioTek, Winooski, VT) at 450 nm. To determine cell viability, chondrocytes were co-cultured with different microspheres (GM@PDA, GM@PDA@EXO, or GM@PDA@S-EXO, 8 mg/well), then washed with PBS and stained using a live/dead staining kit (Beyotime) for 20 min on days 1, 3, and 5. Fluorescence microscopy (Zeiss, Oberkochen, Germany) was used to take photos of live and dead cells.

### **The establishment of OA model in mice**

The animal study protocol was approved by the Ethics Committee of Binzhou Medical College (2023 − 303). Before the surgery, mice were anesthetized with a 2.0% isoflurane and 30% oxygen mixture (RWD Life Sciences, Shenzhen, China). A longitudinal incision was made on the medial side of the knee joint to expose the joint capsule, followed by lateral dislocation of the patella to access the articular cavity. The medial meniscus ligament (MML) was then sectioned using fine scissors. For the sham surgery group, only the joint capsule was exposed, preserving the MML’s structural integrity. Starting one-week post-operation, mice were administered intra-articular injections of GM@PDA, GM@PDA@EXOs, and GM@PDA@S-EXOs microspheres into the joint cavity every two weeks, at 10 µL per injection.

### Histology and immunofluorescence staining

Eight weeks post-surgery, mouse knee joints were harvested and fixed in formalin for 48 h. After decalcification and paraffin embedding, 5 μm thick sagittal sections were prepared from the samples. Histological analysis was performed using hematoxylin and eosin (H&E) and Safranin O-fast green staining. Cartilage degradation was evaluated using the Osteoarthritis Research Society International (OARSI) scoring system. For immunofluorescence staining, sections were blocked with 1% fetal bovine serum (FBS) and permeabilized with 0.1% Triton X-100. After antigen retrieval in a solution heated to 110 °C for 10 min, the sections were incubated overnight at 4 °C with ACAN and SOD3 antibodies, followed by incubation with a secondary antibody (ab6721). The nuclei were stained using DAPI (Beyotime) and subsequently captured using a fluorescence microscope (Zeiss).

### Statistical analysis

Statistical analysis was performed using GraphPad Prism 9.2 software (GraphPad Software Co., Ltd.). The t-test was employed for data that exhibited a normal distribution, with comparisons between two groups conducted using an independent two-tailed Student’s t-test. In instances of multiple group comparisons, one-way analysis of variance (ANOVA) was applied, followed by Tukey’s post hoc test. Statistical significance was determined by a p-value of < 0.05.

## Results

### Analysis of single-cell sequencing results

**Fig. 1:**
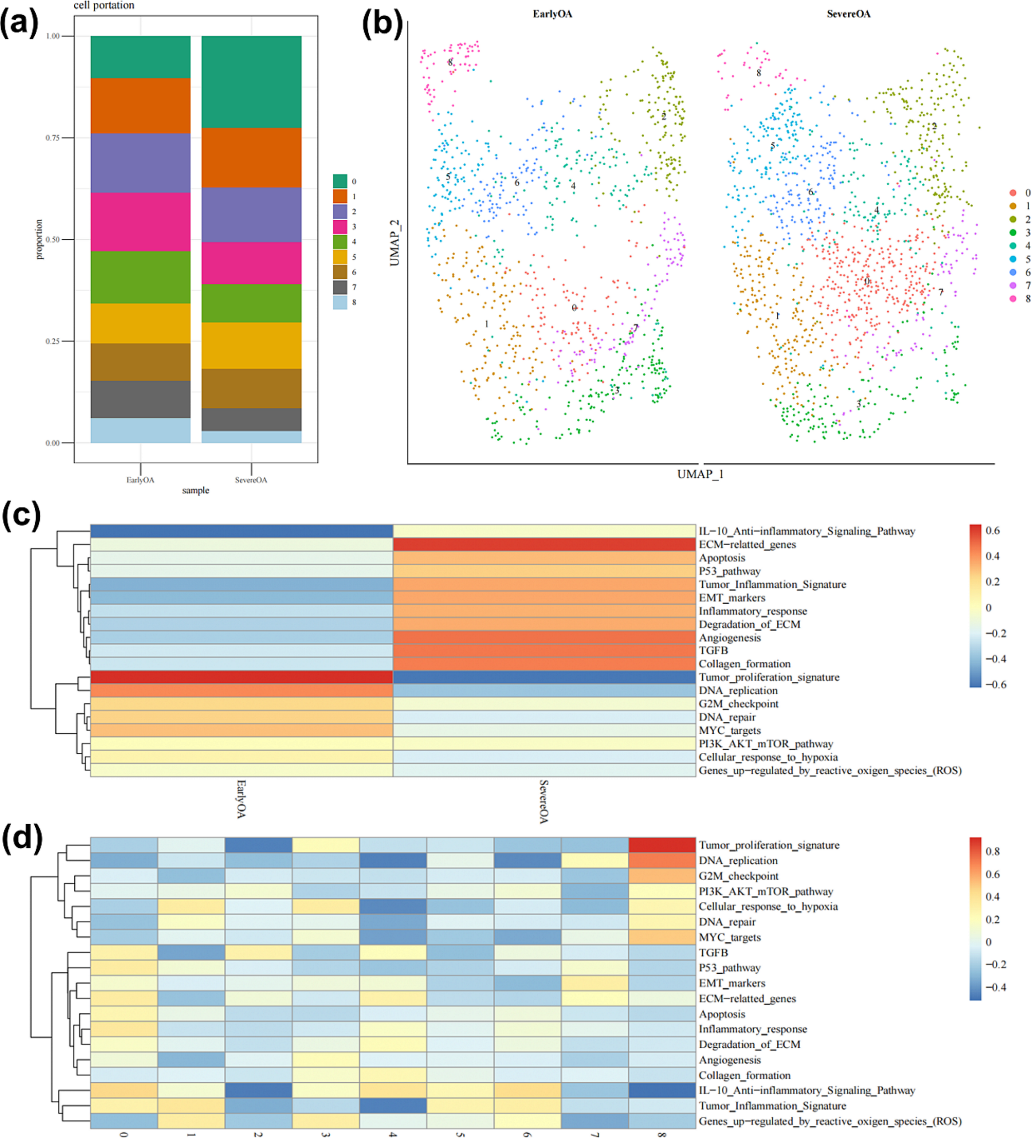
The analysis of single-cell sequencing. (**a**-**b**) Percentage distribution of the 9 different subsets according to either early OA and severe OA. (**c**) Heatmap of significant cellular functions of the synovial fibroblast in early OA and severe OA. (**d**) Heatmap of significant cellular functions for each of the 9 synovial fibroblast subsets

Based on t-SNE analysis, unbiased clustering of cells revealed nine major clusters characterized by distinct gene profiles and typical markers (Fig. 1a, b). In synovial fibroblasts with severe OA, inflammatory responses were markedly elevated, concurrently with a suppression of DNA repair and replication-related pathways. Interestingly, early OA cells exhibited higher expression of genes induced by ROS compared to severe OA cells, indicating a potential defensive feedback mechanism in synovium fibroblasts counteracting OA-induced oxidative stress in the early stages of the disease. In contrast, during advanced OA, the cells’ compensatory and defensive responses were diminished. Examination of different cell clusters showed that clusters 0 (expressing BASP1, SOD2, CTSK, RND3, and GEM) and 3 (expressing IGFBP4, CXCL12, HSPA1A, and SOD3) demonstrated a significant increase in ROS-induced gene expression of LINC01423 compared to the other clusters (Fig. 1c, d).

**Fig. 2:**
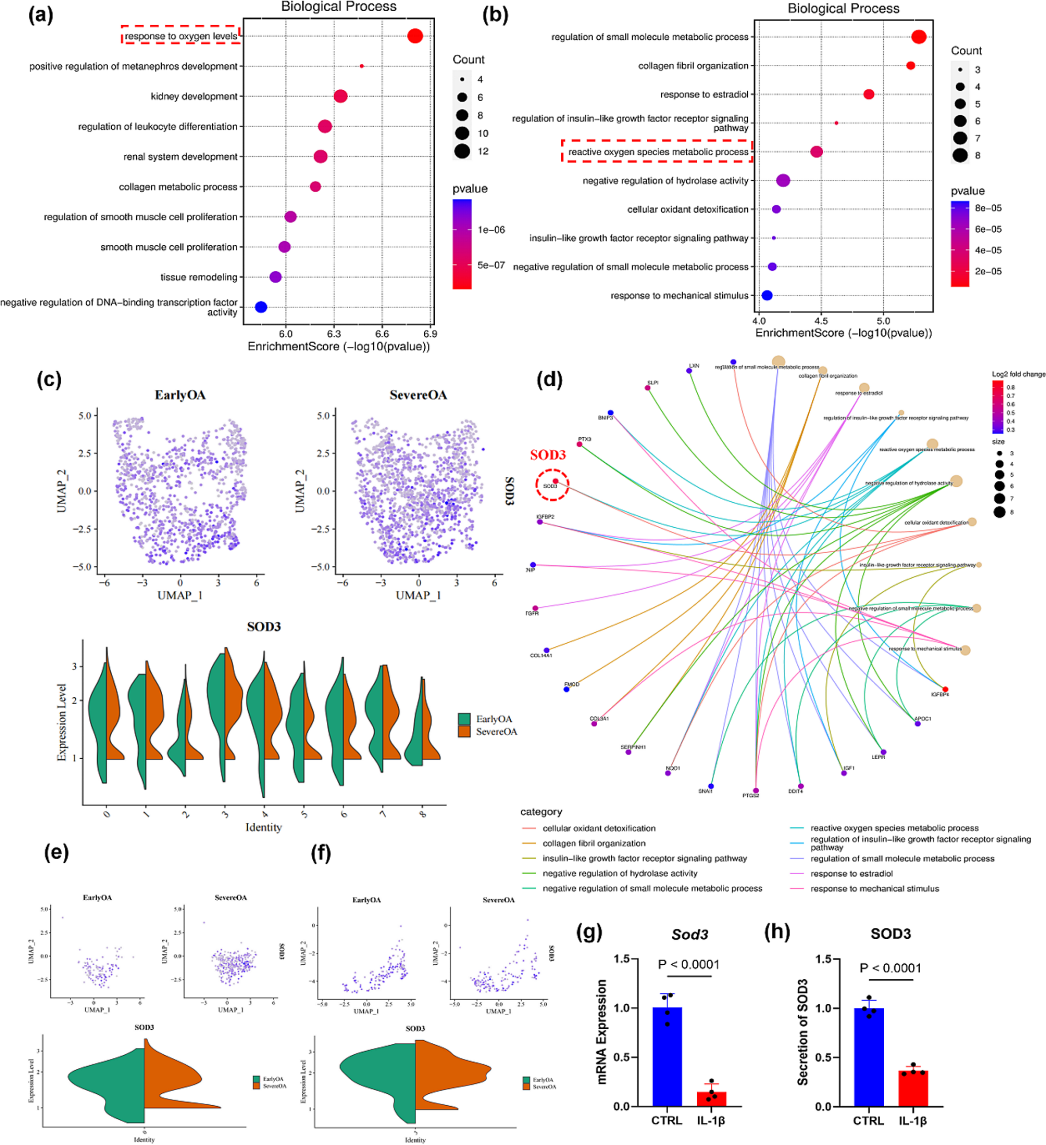
The analysis of antioxidant function of synovial fibroblasts from early OA and severe OA. Biological process bubble diagrams of samples from cluster 0 (**a**) and cluster 3 (**b**) based on GO analysis. (**c**) The difference of SOD3 between early OA and severe OA based on whole-cell population analysis. (**d**) The network diagram of cluster 3. The difference of SOD3 between early OA and severe OA based on cluster 0 (**e**) and cluster 3 (**f**) population analysis. (**g**) RT-PCR analysis for the mRNA expression of *Sod3*, *n* = 4. (**h**) Elisa analysis for the secretion of SOD3, *n* = 4

Gene ontology (GO) analysis of clusters 0 and 3 indicated a significant upregulation in antioxidant-related pathways within these clusters (Fig. 2a, b). Two isoforms within the SOD family, SOD2 and SOD3, were identified as crucial contributors to this mechanism. The network diagrams for clusters 0 and 3 illustrate the involvement of both SOD2 and SOD3 in pathways countering oxidative stress (Fig. 2d, Supplementary Fig. 1). Analyses of the whole-cell population revealed an increase in SOD2 and SOD3 expression in synovial fibroblasts from severe OA cases, indicating an intrinsic upregulation of SODs as a defensive response to inflammation and heightened ROS levels (Fig. 2c, Supplementary Fig. 2). Findings from clusters 0 and 3 were consistent with these observations (Fig. 2e, f, Supplementary Figs. 3,4). IL-1β exposure resulted in a reduced gene expression level of SOD3 in chondrocytes, a finding corroborated by enzyme-linked immunosorbent assay results, which demonstrated a marked decrease in SOD3 content (*P* < 0.0001) (Fig. 2g, h). Similarly, SOD2 gene expression was diminished in chondrocytes treated with IL-1β (*P* = 0.0014) (Supplementary Fig. 5).

### Characterization of S-EXO nanoparticles and verification of its antioxidant function

**Fig. 3:**
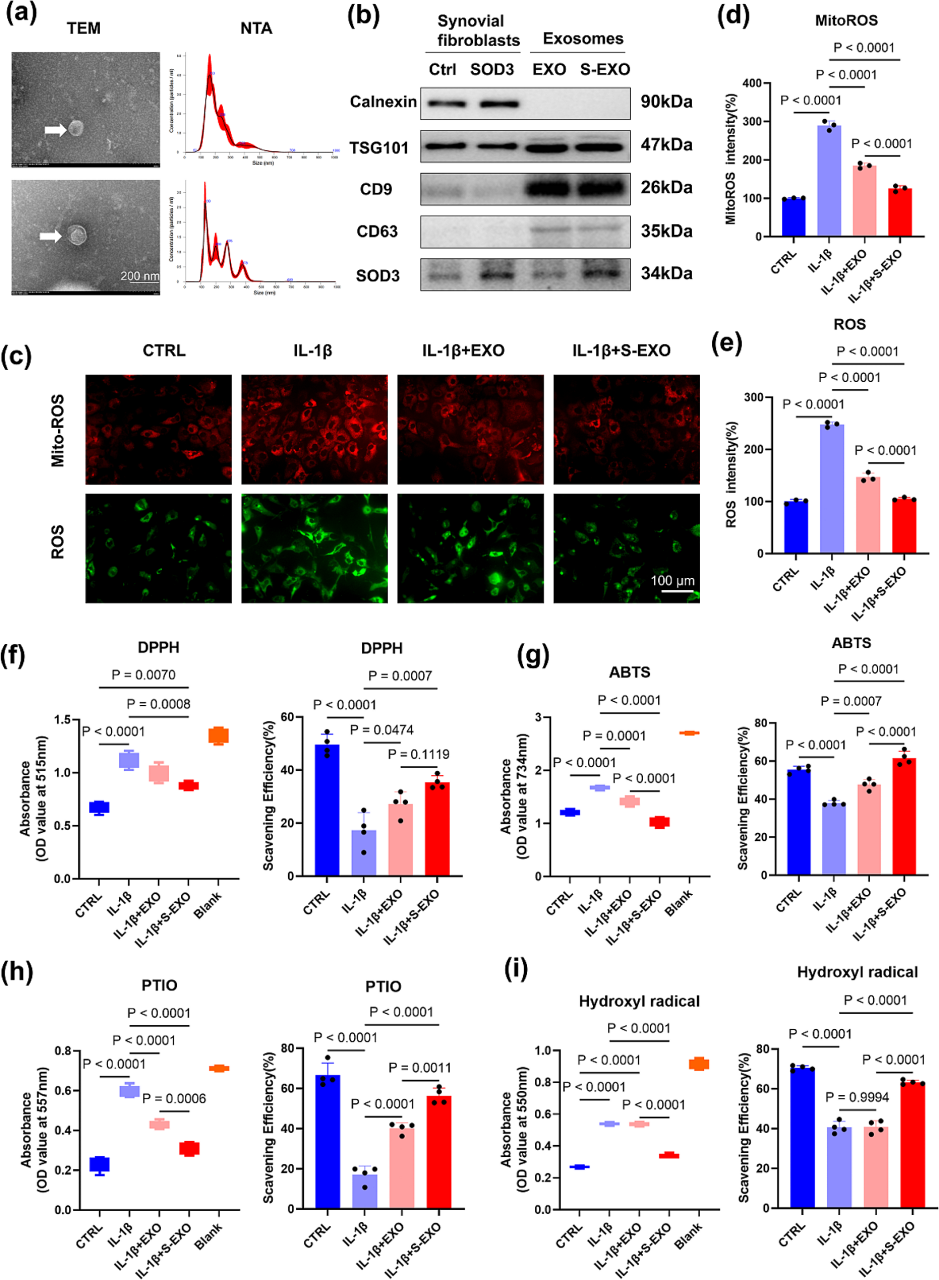
S-EXOs enhanced the antioxidant capacity of chondrocytes in inflammatory conditions induced by IL-1β. (**a**) Morphology of EXOs and S-EXOs captured by TEM and the particle size distribution detected by NTA. Scale bar = 200 nm, *n* = 3. (**b**) Western blot analysis of the EXOs and S-EXOs specific protein markers, *n* = 3. (**c**-**e**) The effects of EXOs and S-EXOs on the amount of mitoROS (Red) and intracellular ROS (Green) in chondrocytes, *n* = 3. (**f**) DPPH, (**g**) ABTS, (**h**) PTIO and (**i**) hydroxyl radical-scavenging ability of the EXOs and S-EXOs detected via UV − vis spectroscopy, *n* = 4

TEM analysis showed that the circular convex structures of EXO and S-EXO nanoparticles possess characteristic exosomal features [27]. NTA confirmed that the size of these cellular particles fell within the nanometer range, with no notable differences between the groups (Fig. 3a). Both nanoparticle types were positively marked for TSG101, CD63, and CD9. The enhanced expression of SOD3 in synovial fibroblasts was confirmed (*P* = 0.0016) (Supplementary Fig. 6), and a significant increase in SOD3 content was observed in exosomes from these fibroblasts (*P* = 0.0007) (Fig. 3b, Supplementary Fig. 6). In comparison to the CTRL group, the administration of IL-1β resulted in a 191.1% increase in mitoROS and a 147.7% increase in ROS levels. Each exosome type demonstrated an ability to boost chondrocytes’ antioxidant capacity, with the EXO group showing a notable decrease of 104.6% (*P* < 0.0001) in mitoROS and 101.2% (*P* < 0.0001) in ROS levels compared to the IL-1β-treated group. The S-EXO group exhibited even greater reductions, 164.2% (*P* < 0.0001) in mitoROS and 142.7% (*P* < 0.0001) in ROS levels, indicating a superior antioxidant effect compared to the EXO group (*P* < 0.0001) (Fig. 3c, e).

Assays involving DPPH, ABTS+, PTIO, and hydroxyl free radicals indicated that exosomes from synovioblasts could enhance chondrocytes’ free radical scavenging capacity. In the DPPH assay, ROS clearance rates for EXO and S-EXO groups increased by 57.0% (*P* = 0.0474) and 104.3% (*P* = 0.0007), respectively, against the IL-1β group (Fig. 3f). ABTS + assay results showed clearance rate improvements of 25.8% (*P* = 0.0007) for EXO and 62.6% (*P* < 0.0001) for S-EXO compared to the IL-1β group, with S-EXO significantly outperforming EXO (*P* < 0.0001) (Fig. 3g). PTIO assays revealed increases of 134.9% (*P* < 0.0001) for EXO and 2.3-fold (*P* < 0.0001) for S-EXO in clearance rates compared to the IL-1β group, with S-EXO showing enhanced effectiveness over EXO (*P* = 0.0011) (Fig. 3h). In hydroxyl free radical detection, only S-EXO significantly increased the clearance rate by 55.7% (*P* < 0.0001) (Fig. 3i).

### S-EXO nanoparticles protected the cartilage extracellular matrix (ECM)

**Fig. 4:**
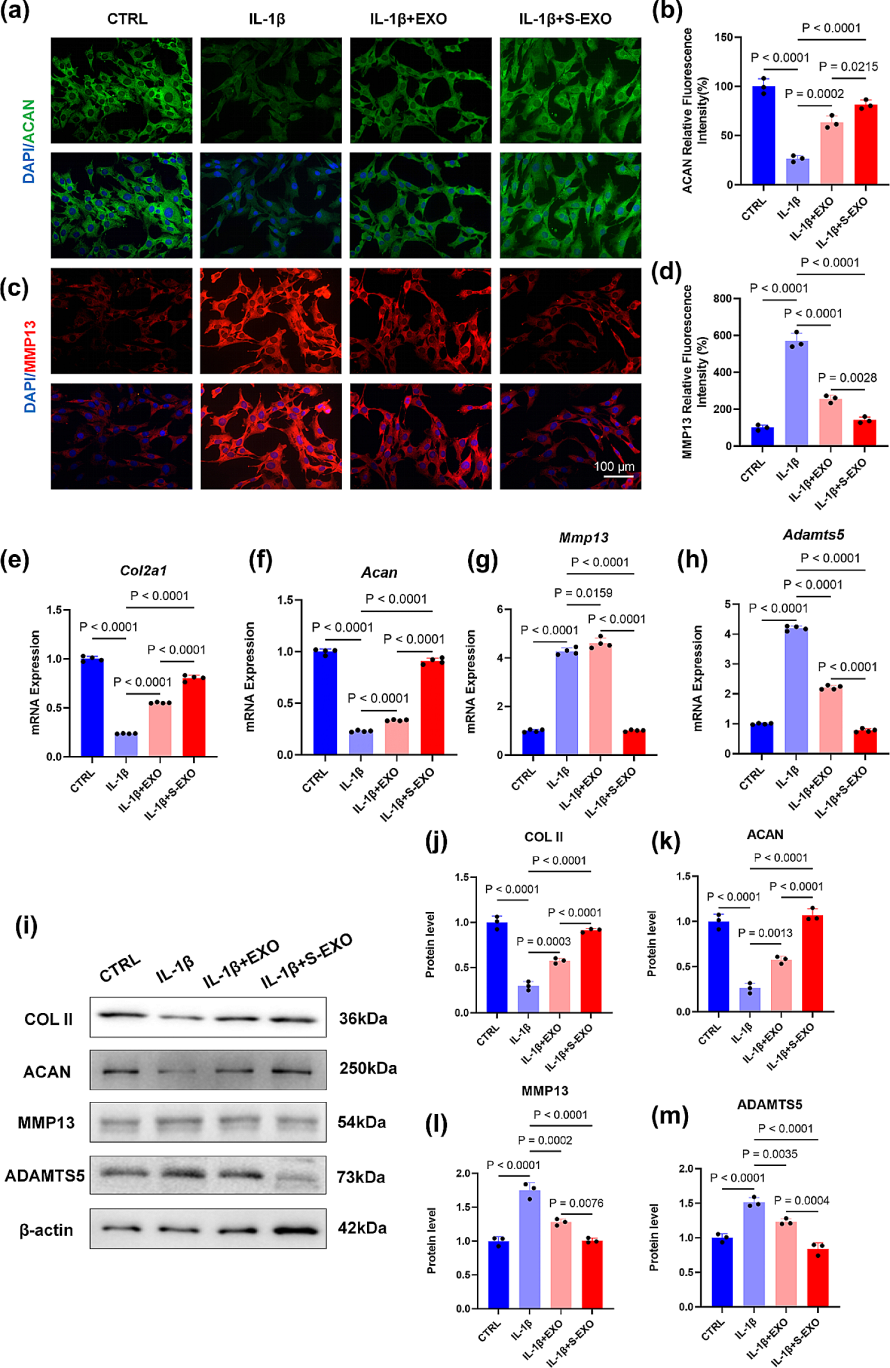
The effects of EXOs and S-EXOs on ECM metabolism of chondrocytes in inflammatory micro-environment induced by IL-1β. (**a**-**b**) IF analysis for the expression of ACAN. (**c**-**d**) IF analysis for the expression of MMP13. Scale bar = 100 μm, *n* = 3. (**e**-**h**) RT-PCR analysis for the mRNA expression of *Col2a1, Acan, Mmp13 and Adamts5*, *n* = 4. (**i**-**m**) The protein levels of COL II, ACAN, MMP13 and ADAMTS5, *n* = 3

To elucidate the effect of S-EXO nanoparticles on cartilage matrix synthesis, immunofluorescence staining was employed. Compared to the IL-1β-treated group, both EXO and S-EXO groups showed marked increases in ACAN expression, a crucial ECM component, by 139.5% (*P* = 0.0002) and 2.07-fold (*P* < 0.0001), respectively. Notably, ACAN expression in the S-EXO group was significantly higher than in the EXO group (*P* = 0.0215) (Fig. 4a, b). Additionally, there was a 55.2% (*P* < 0.0001) and 75.3% (*P* < 0.0001) reduction in MMP13 expression for the EXO and S-EXO groups, respectively, with the S-EXO group showing a significant decrease compared to the EXO group (*P* = 0.0028) (Fig. 4c, d).

Treatment with these nanoparticles led to an upregulation of ECM synthesis-related genes. The EXO group exhibited a 132.8% (*P* < 0.0001) increase in *Col2a1* expression and a 43.6% (*P* < 0.0001) increase in *ACAN* expression compared to the IL-1β group, while the S-EXO group showed a 2.4-fold (*P* < 0.0001) and 43.6% (*P* < 0.0001) increase in these genes, respectively. The S-EXO group significantly outperformed the EXO group in enhancing *Col2a1 *and *ACAN* expression (*P* < 0.0001) (Fig. 4e, f). Moreover, S-EXO nanoparticles reduced the expression of matrix degradation-related genes, *Mmp13* and *Adamts5*, by 76.3% (*P* < 0.0001) and 81.1% (*P* < 0.0001), respectively (Fig. 4g, h). Western blot analysis further confirmed that, relative to the IL-1β group, S-EXO treatment significantly increased COL II and ACAN protein levels by 2.1-fold (*P* < 0.0001) and 3.1-fold (*P* < 0.0001), respectively, and decreased MMP13 and ADAMTS5 expression by 42.3% (*P* < 0.0001) and 44.4% (*P* < 0.0001) (Fig. 4i-m). When compared to the EXO group, the S-EXO group showed substantial increases in COL II and ACAN levels (*P* < 0.0001), along with significant reductions in MMP13 (*P* = 0.0076) and ADAMTS5 (*P* = 0.0004) (Fig. 4i-m).

### Characterization and biocompatibility of GM@PDA@S-EXOs

**Fig. 5:**
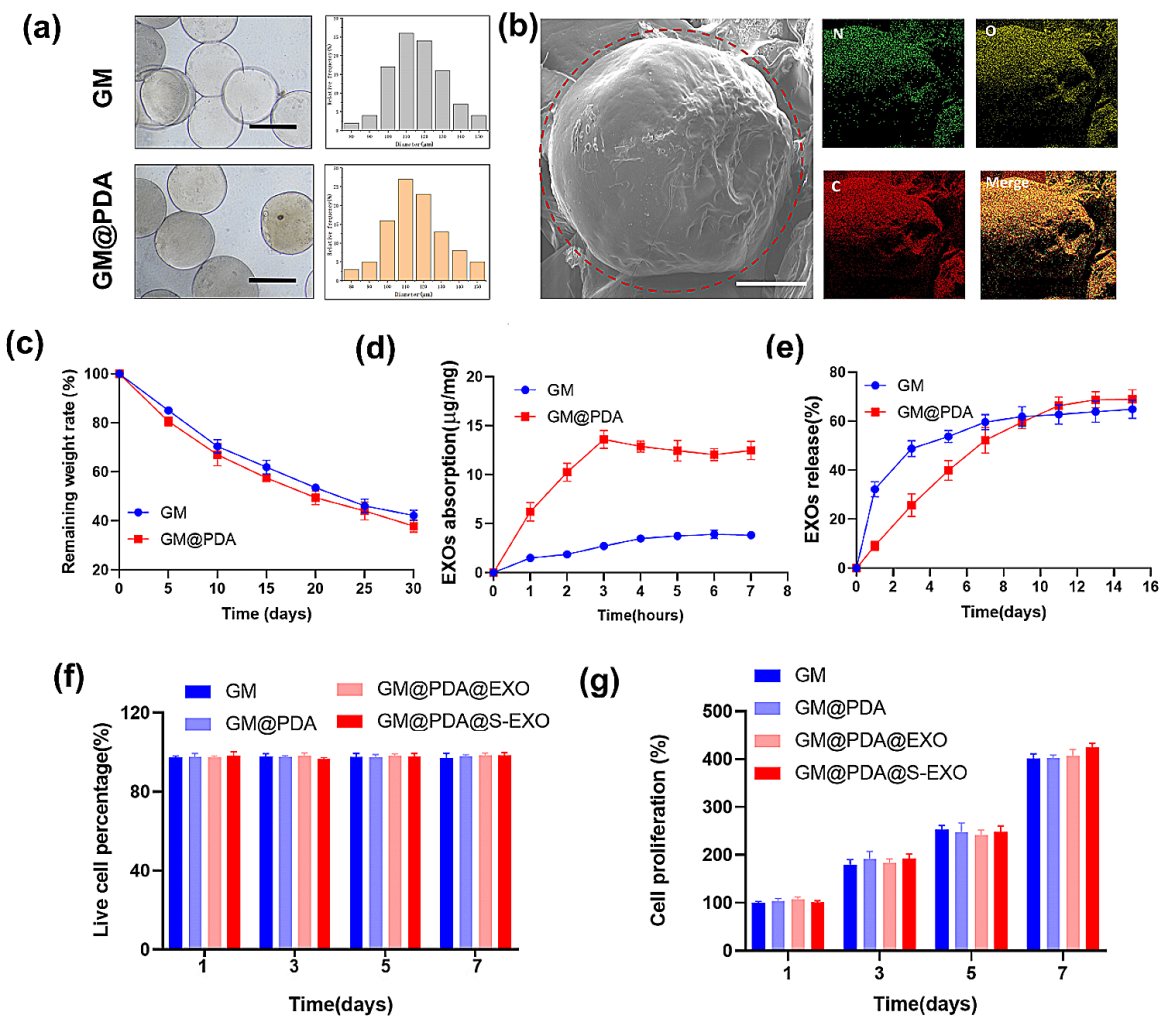
Characterization of EXOs bearing microspheres. (**a**) The gross view and diameter distribution of microspheres. Scale bar = 100 μm. (**b**) The representative SEM and EDS images of dispersed microspheres. Scale bar = 20 μm. (**c**) The rate of remaining weight, *n* = 3. (**d**) The loading efficiency curves for the EXOs-loaded microspheres, *n* = 3. (**e**) Release profiles of EXOs from the EXOs-loaded microspheres, *n* = 3. (**f**) The quantitative analysis of Live&dead cells cultured on various microspheres, *n* = 3. (**g**) The cell proliferation analysis of the cells cultured on various microspheres using CCK-8, *n* = 3

The GM microspheres were white, while those modified with PDA (GM@PDA) appeared light-yellow. The monodisperse microspheres maintained a spherical shape, with an average size of 119.5 μm for GM and 124.1 μm for GM@PDA (Fig. 5a). SEM and elemental mapping analyses identified nitrogen signals (shown in green) on the GM@PDA surface, confirming successful PDA coating (Fig. 5b). The degradation rates of both microsphere types were comparable.

After 30 days, the residual mass was 42.2% for GM and 37.9% for GM@PDA microspheres (Fig. 5c). PDA modification enhanced the loading capacity of GM@PDA microspheres to 13.6 µg/mg within three hours, whereas GM microspheres reached a peak loading rate of 3.9 µg/mg after six hours (Fig. 5d). Exosome release from GM microspheres occurred swiftly within three days, with no significant release after one week. In contrast, GM@PDA microspheres exhibited a slower release over two weeks (Fig. 5e). Live/dead cell analysis showed high chondrocyte viability with few dead cells in all groups, indicating negligible cytotoxicity from the microspheres (Fig. 5f). Furthermore, CCK-8 assays demonstrated that neither type of microsphere significantly affected cell proliferation (Fig. 5g).

### Intra-articular injection of GM@PDA@S-EXO alleviated the OA progression via increasing the amount of SOD3

**Fig. 6:**
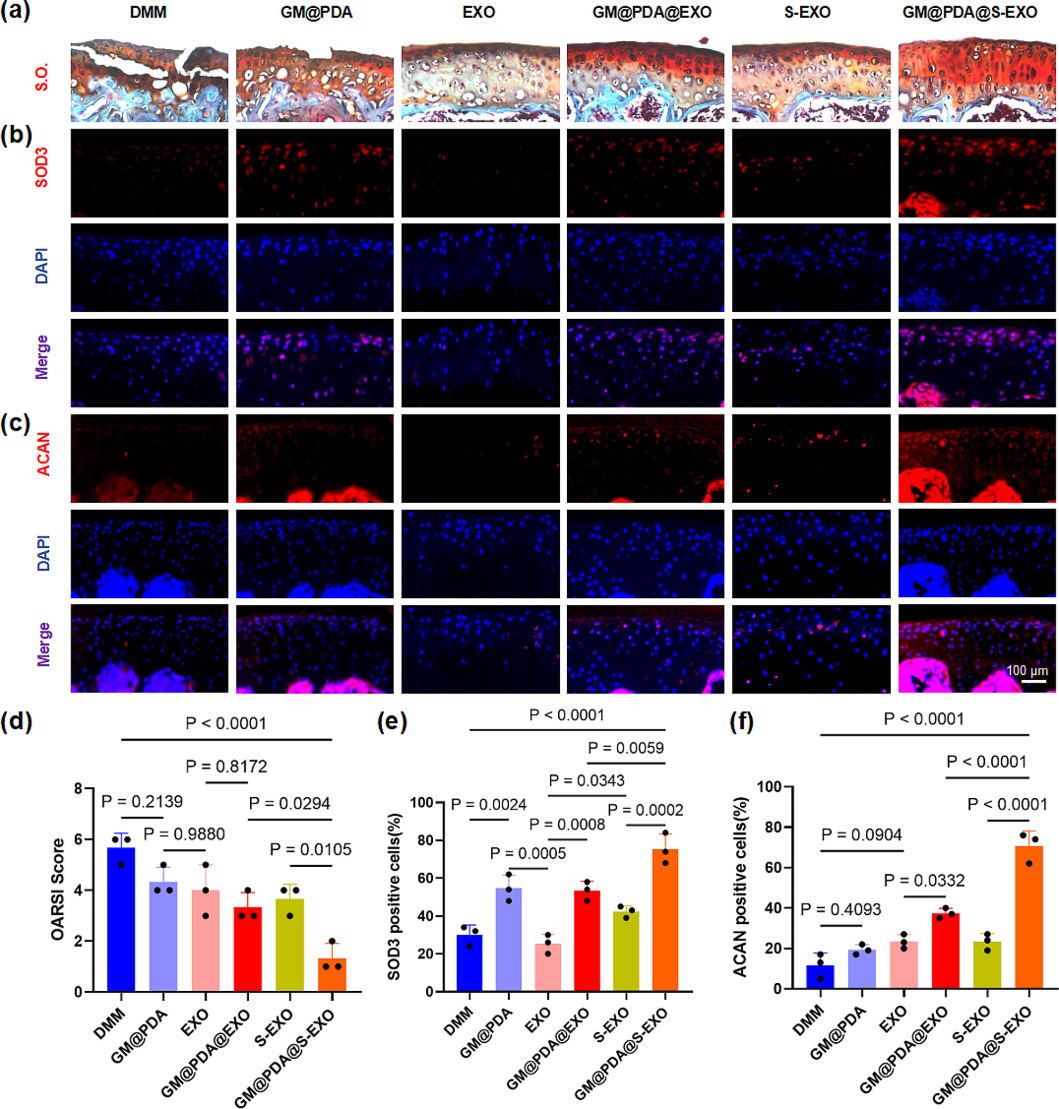
EXOs-loaded microspheres alleviated the progress of OA by delivering SOD3. (**a**) Representative images of cartilage stained by Safranin O-fast green (S.O.). (**b**&**e**) IF analysis for the expression of SOD3. (**c**&**f**) IF analysis for the expression of ACAN. (**d**) OARSI scores. Scale bar = 100 μm, *n* = 3

This study assessed the effect of GM@PDA@S-EXO on cartilage degeneration in vivo. Safranin O-fast green staining revealed that intra-articular injection of GM@PDA@S-EXO microspheres preserved more glycosaminoglycan expression in the articular cartilage (Fig. 6a). Additionally, GM@PDA@S-EXO treatment was associated with a reduction in the OARSI score, indicating an alleviation of OA severity. Compared to the free S-EXO, the OARSI score of the GM@PDA@S-EXO group decreased by 63.6% (*P* = 0.0105) (Fig. 6d). IF analysis demonstrated that GM@PDA@EXO and GM@PDA@S-EXO treatment significantly increased the expression of SOD3 in cartilage of DMM-induced OA mice. The expression level of SOD3 in the GM@PDA@S-EXO group increased by 78.0% compared to the free S-EXO group (*P* = 0.0002) (Fig. 6b&e). Furthermore, GM@PDA@EXO and GM@PDA@S-EXO treatment increased the expression of ACAN compared to the DMM group. The expression level of ACAN in the GM@PDA@S-EXO group increased by 2.0-fold compared to the free S-EXO group (*P* < 0.0001) (Fig. 6c&f).

## Discussion

In this study, we identified SOD3 as a crucial protein through single-cell sequencing analysis, noting its enhancement of chondrocytes’ antioxidant capabilities and its beneficial effects on cartilage matrix metabolism in vitro. Following this, hydrogel microspheres encapsulating SOD3 were injected into the joint cavity of OA mice, leading to a delay in OA progression. The synovium plays a vital role in maintaining cartilage homeostasis by producing and secreting various molecules [8]. In particular, synoviocytes in the intimal layer are responsible for synthesizing synovial fluid, which lubricates the joint and provides nutrients to the cartilage [28]. SOD3, secreted by synovial fibroblasts, is conveyed to the cartilage via joint fluid, where it is believed to exert a protective role [29]. Our examination of SOD3 levels in chondrocytes within an IL-1β-induced inflammatory milieu revealed a reduction in SOD3 expression as inflammation escalated, contrasting with the increase in SOD3 expression in synovial fibroblasts. These findings led us to hypothesize that synovial fibroblasts secrete SOD3, which then navigates to the cartilage amidst inflammation and ROS, thereby assisting chondrocytes in combating oxidative stress. This hypothesis aligns with previous research showing the impact of various synovial factors, including TGF-β and IGF-1, on cartilage and chondrocytes, particularly in terms of modulating inflammatory responses and matrix metabolism [8]. Building on these foundational insights, we propose a novel pathway wherein SOD3, an exocrine protein from synovial fibroblasts, influences chondrocytes and modulates matrix metabolism by adjusting the chondrocyte oxidative microenvironment.

SOD3, an extracellular antioxidant enzyme, efficiently converts superoxide free radicals into hydrogen peroxide, thus preventing the accumulation of ROS [30]. Mice deficient in SOD3 exhibited increased synovial inflammation, cartilage degradation, and bone erosion, whereas SOD3 overexpression mitigated these effects, reducing disease severity [31]. Beyond neutralizing ROS, SOD3 modulates inflammatory signaling pathways, demonstrated by its capacity to decrease the secretion of pro-inflammatory cytokines like IL-1β, IL-2, IL-4, IFN-γ, and TNF-α [32]. Furthermore, SOD3 administration has been demonstrated to inhibit mononuclear cell infiltration and synovial inflammatory cell proliferation in an OA model [33]. Treatment with a synthetic SOD3 mimetic significantly reduced nitrotyrosine expression, a marker of peroxynitrite formation, in inflamed joints [34]. SOD3 also inhibits the activation of nuclear factor κB (NF-κB) [35], a transcription factor involved in producing pro-inflammatory cytokines and MMPs [36]. To counteract OA-induced cartilage degeneration, we aimed to obtain synovial fibroblast-derived SOD3 with sustained biological activity and high concentration. We achieved this by overexpressing SOD3 in cells, then isolating SOD3-rich exosomes (S-EXO) from the supernatant. Exosomes offer multiple advantages in OA treatment, their small size (30–150 nm) enables easy penetration of tissue and biological barriers, making them ideal for delivering therapeutic agents to target cells. With their low immunogenicity, high biocompatibility, and efficacy, exosomes serve as an effective bioactive factor delivery system [37]. Their inherent stability allows for extended storage without losing biological activity [38]. Zhao et al. demonstrated that intra-articular injection of MSC-derived exosomes reduced joint inflammation and cartilage damage in OA rats, achieving targeted miR-199a-3p delivery to chondrocytes and activating mTOR-related pathways that regulate chondrocyte autophagy and apoptosis [39]. In our in vitro study, S-EXOs were used to address the reduced antioxidant capacity and matrix metabolism of chondrocytes in an inflammatory environment. The results showed that S-EXOs had a more substantial therapeutic effect compared to conventional EXOs, supporting our hypothesis and offering a new approach for SOD3 extraction and application.

Hydrogel microspheres are increasingly acknowledged as a viable therapeutic strategy for osteoarthritis (OA) treatment due to their unique features and advantages [40]. The reduction of lubricating proteins on OA cartilage surfaces can increase friction, exacerbating OA progression [41]. Hydrogel microspheres can act as lubricants in the articular cavity, alleviating OA symptoms and slowing cartilage degradation [42]. Importantly, they enable a controlled and sustained release of therapeutic agents, including anti-inflammatory and disease-modifying drugs [40]. This mechanism allows for extended drug presence at the inflammation site, reducing administration frequency and minimizing systemic side effects. Hydrogel microspheres can encapsulate both hydrophilic and hydrophobic drugs, improving their solubility and stability [43]. Their small size allows penetration into the joint space, ensuring effective drug delivery to target tissues. Thus, microspheres offer a dual function as a drug delivery system and a therapeutic agent with lubricating properties [44]. In our research, gelatin microspheres were modified with PDA to enhance exosome adsorption, enabling sustained S-EXOs release in the body. This highlights the practicality, versatility, and effectiveness of microspheres in OA therapy. Jin et al. adopted a methodology akin to ours for exosome delivery, encapsulating stem cell-derived exosomes from human deciduous teeth in hydrogel microspheres [27]. Their bioinformatics analysis indicated these exosomes, rich in anti-aging signals, could reverse aging traits in aged tendon stem cells and promote their differentiation into tenocytes by altering histone methylation and inhibiting NF-κB. Following this, they administered these exosomes locally using hydrogel microspheres. In parallel, our in vitro study utilized S-EXOs to tackle reduced antioxidant capacity and stromal metabolism in chondrocytes under inflammatory conditions, finding that S-EXOs had more significant therapeutic effects than conventional EXOs. This not only confirmed our initial hypothesis but also offered a new approach to the extraction and application of SOD3. Our in vivo studies demonstrated that microspheres can precisely deliver S-EXOs to articular cartilage and significantly mitigate OA progression. These microspheres ensure targeted drug delivery while reducing friction and lubricating the joint cavity. However, the study has limitations, particularly the lack of proteomic sequencing for exosomes, which would have yielded a deeper insight into the therapeutic components involved. Additionally, the microspheres and exosomes lack specific cartilage-targeting capabilities, which may affect the effectiveness of protein delivery. Future research will explore the molecular mechanisms in more depth and improve the drug delivery system by integrating cartilage-targeting peptides to enhance delivery efficiency and accuracy.

## Conclusion

Analysis of single-cell sequencing data has identified SOD3 as a novel potential target for the synovial regulation of cartilage. This target enhances the antioxidant capabilities of chondrocytes and protects cartilage matrix metabolism. Furthermore, intra-articular administration of microspheres containing SOD3-enriched exosomes has been demonstrated to effectively mitigate OA by increasing SOD3 levels. This study introduces a novel approach for exosome functionalization and presents a promising strategy for OA treatment.

### Electronic supplementary material

Below is the link to the electronic supplementary material.

Supplementary Material 1

## Data Availability

The data that supports the findings of this study are available in the supplementary material of this article.
