# Supplementary material for: Bio-nanoparticles loaded with synovial-derived exosomes ameliorate osteoarthritis progression by modifying the oxidative microenvironment
Source: J Nanobiotechnology. 2024 May 20;22:271. doi: 10.1186/s12951-024-02538-w (PMC11103857; doi:10.1186/s12951-024-02538-w)
Supplement: Supplementary file 1 — Supplementary Material 1 [file 12951_2024_2538_MOESM1_ESM.docx]

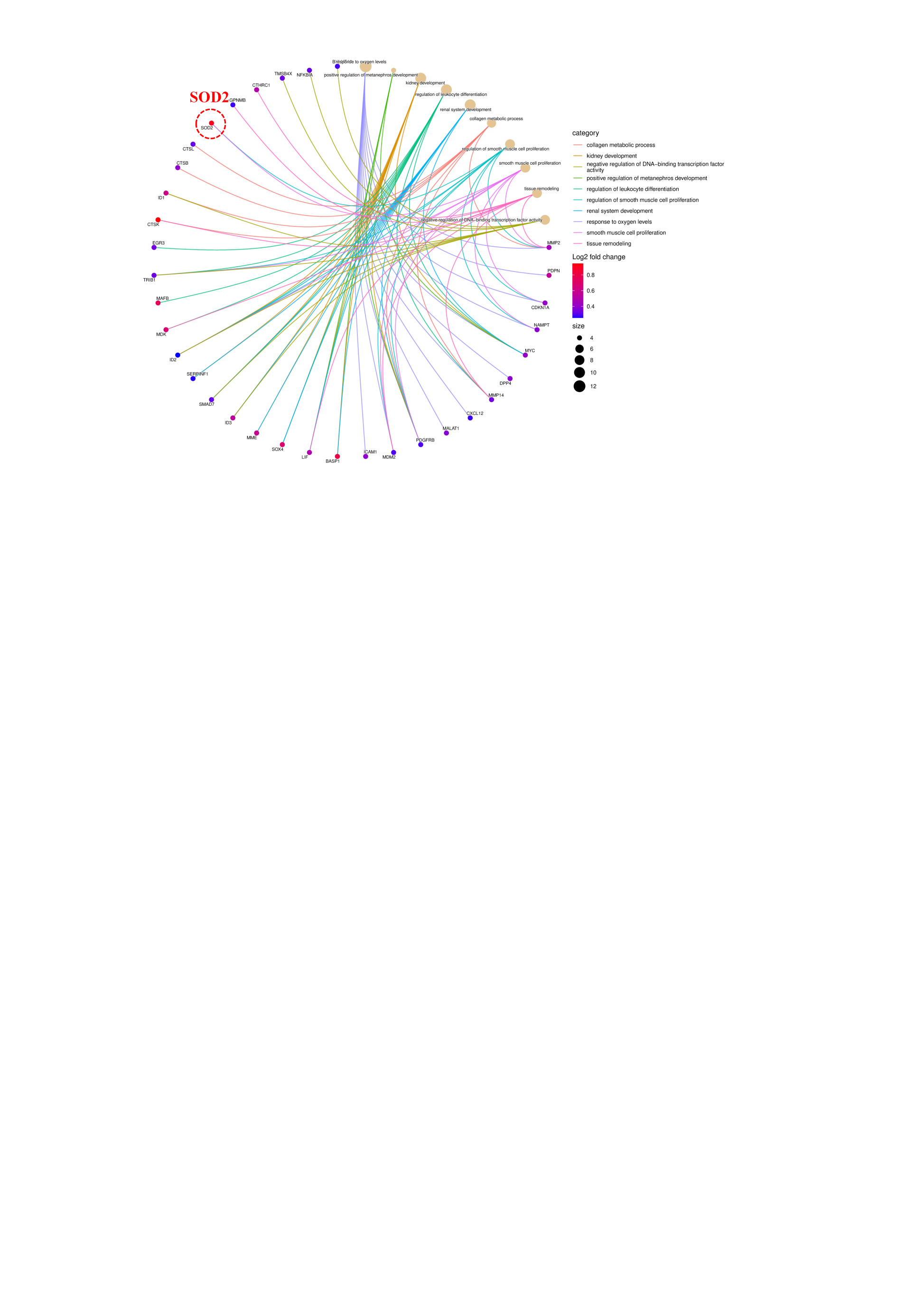


**Supplementary Fig. 1** The network diagram of cluster 0.


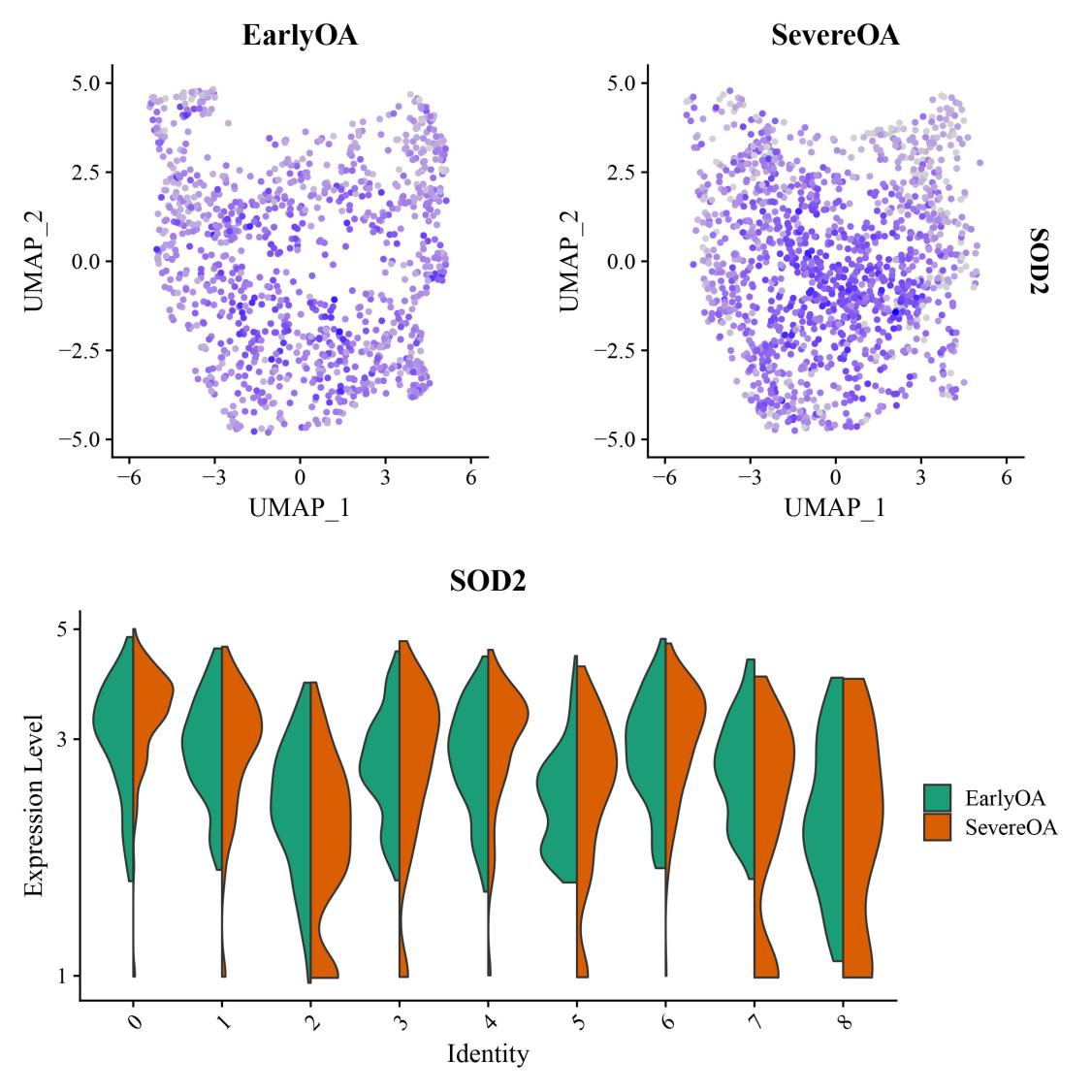


**Supplementary Fig. 2** The difference of SOD2 between early OA and severe OA based on whole-cell population analysis.


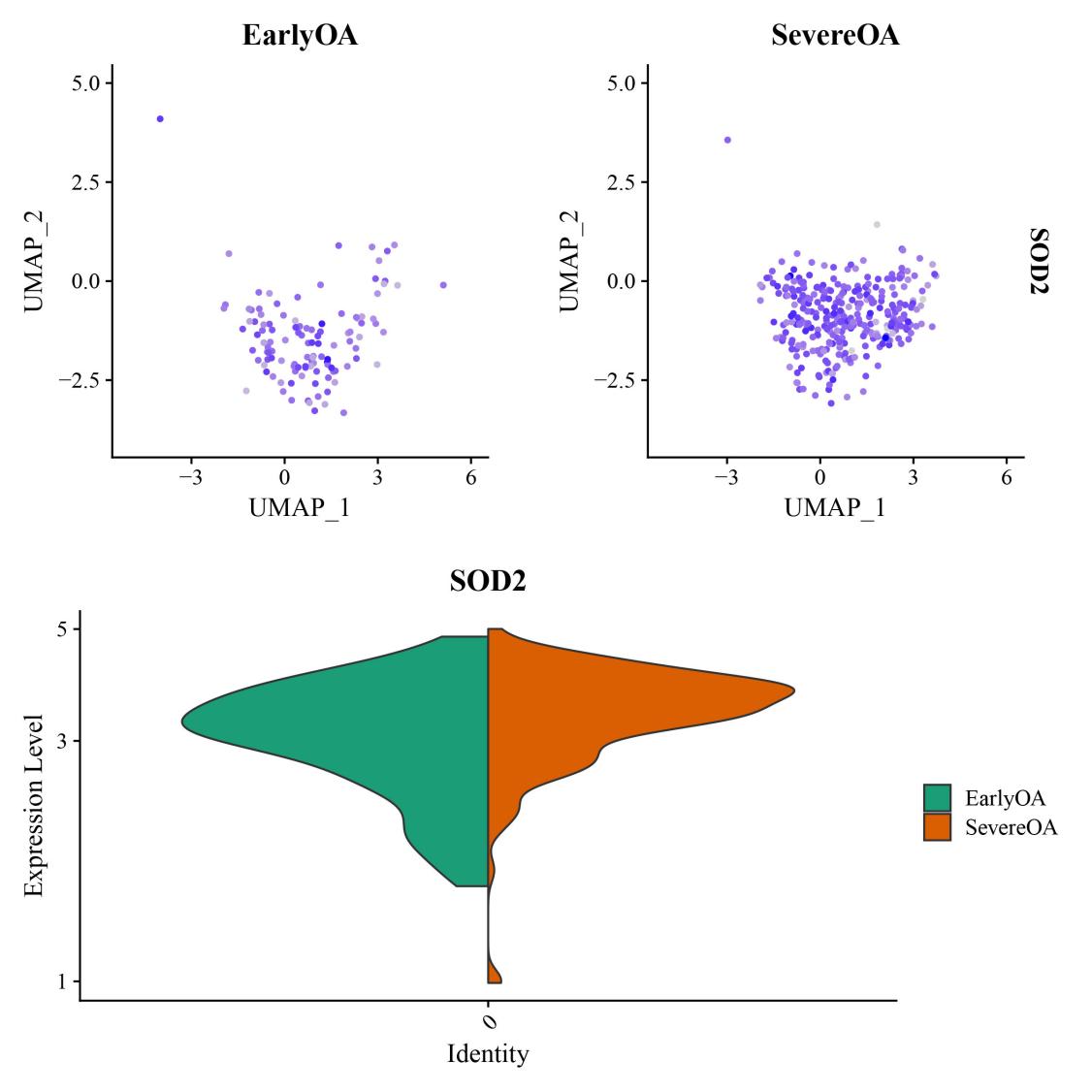


**Supplementary Fig. 3** The difference of SOD2 between early OA and severe OA based on cluster 0 population analysis.


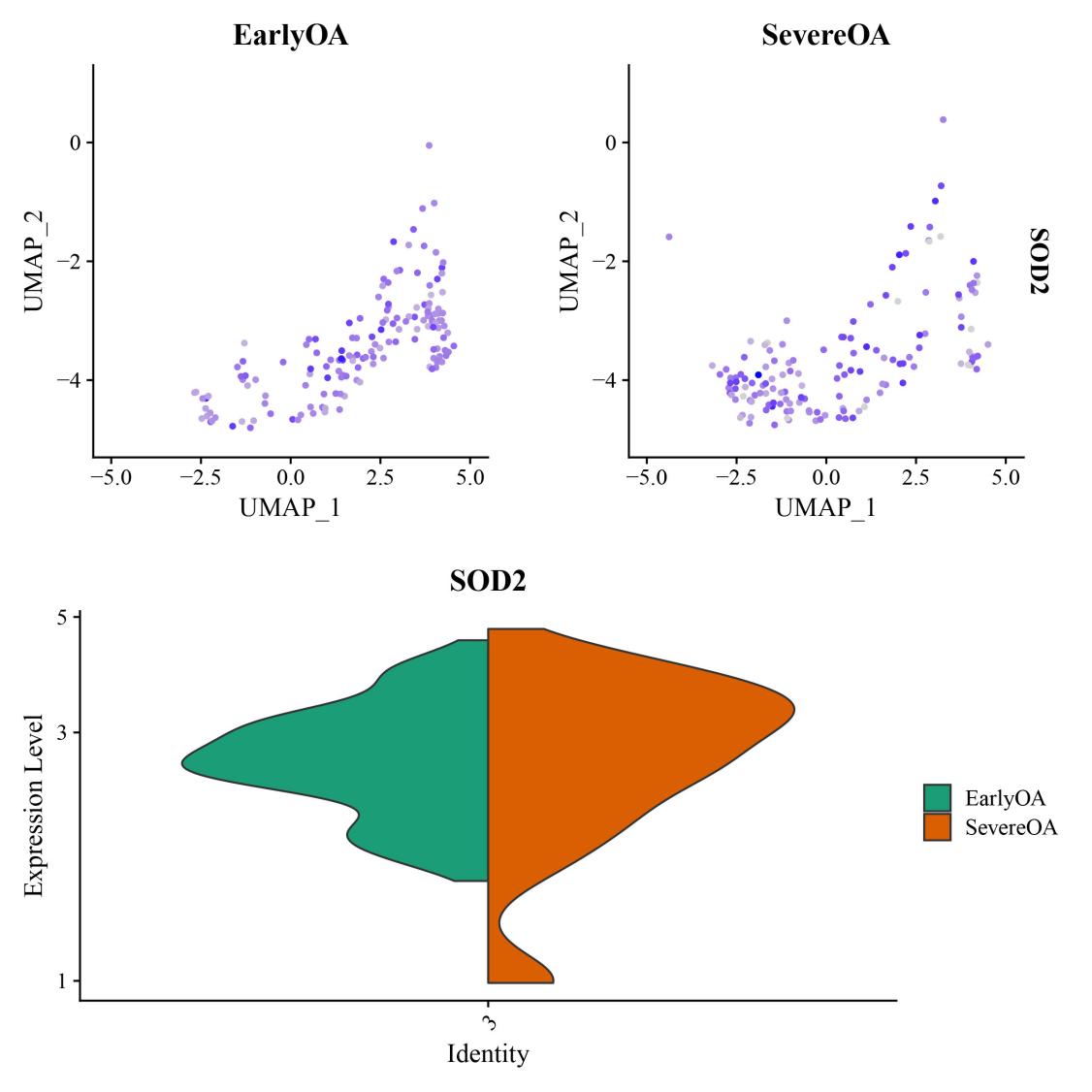


**Supplementary Fig. 4** The difference of SOD2 between early OA and severe OA based on cluster 3 population analysis.


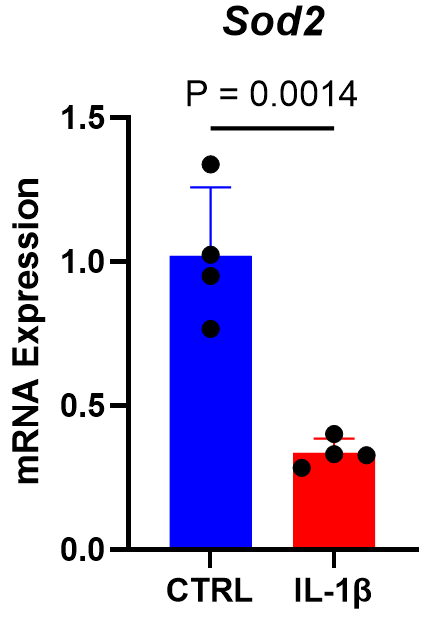


**Supplementary Fig. 5** The gene expression of Sod2, n=4.


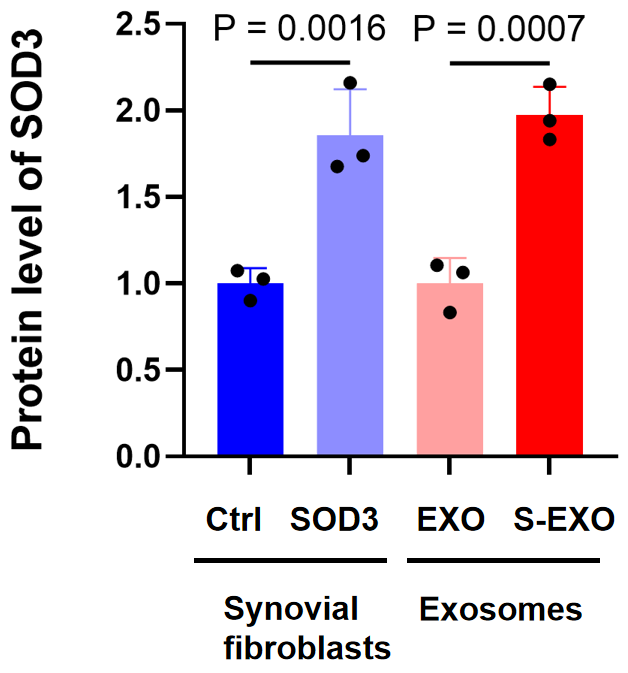


**Supplementary Fig. 6** The quantitative analysis of SOD3 protein, n=3.

**Supplementary table 1:** The primer used in Sod3 mimics.

| Gene | Primer (5'-3') |
| --- | --- |
|  |  |
| SOD3 Mouse | ATGTTGGCCTTCTTGTTCTACGGCTTGCTACTGGCGGCCTGTGGCTCTGTCACCATGTCAAATCCAGGGGAGTCCAGCTTCGACCTAGCAGACAGGCTTGACCCGGTTGAGAAGATAGACAGGCTTGACCTGGTTGAGAAGATAGGCGACACGCATGCCAAAGTGCTGGAGATCTGGATGGAGCTAGGACGACGAAGGGAGGTGGATGCTGCCGAGATGCATGCAATCTGCAGGGTACAACCATCAGCCACGCTGCCACCGGATCAGCCGCAGATCACCGGCTTGGTTCTCTTCCGGCAGCTGGGGCCGGGCTCCAGGCTTGAGGCCTATTTCAGTCTGGAGGGCTTCCCAGCTGAGCAGAACGCCTCCAACCGTGCCATCCACGTGCATGAGTTCGGGGACCTGAGCCAGGGCTGCGATTCCACCGGGCCGCACTACAACCCGATGGAGGTGCCGCACCCTCAGCACCCGGGCGACTTTGGCAACTTCGTGGTGCGCAACGGCCAGCTCTGGAGGCATCGCGTCGGCCTGACCGCGTCGCTGGCCGGACCGCACGCCATCTTGGGCCGCTCTGTGGTGGTCCACGCCGGCGAGGACGACCTGGGTAAAGGTGGCAACCAGGCCAGCCTGCAGAACGGCAATGCAGGTCGCCGGCTCGCCTGCTGCGTGGTAGGCACCAGCAGCTCCGCCGCCTGGGAGAGCCAGACAAAGGAGCGCAAGAAGCGGCGGCGGGAGAGCGAGTGCAAGACCACTTAA |

**Supplementary table 2:** The primer used in RT-PCR.

| adamts5-F | AACAGGAGGATCATCGCAGATACAG |
| --- | --- |
| adamts5-R | GACTCGTGGTGACAATGACATGAAG |
| mmp13-F | TCATACTACCATCCTGCGACTCTTG |
| mmp13-R | TGCCAGTCACCTCTAAGCCAAAG |
| acan-F | ATTGAGGTCTGTGCCATCTGTGAG |
| acan-R | CTACCCAGTCCAGCCGAGAAATG |
| col2a1-F | CGCTACACTCAAGTCACTGAACAAC |
| col2a1-R | CAATCCAGTAGTCTCCGCTCTTCC |
| Sod2-F | CAGACCTGCCTTACGACTATGG |
| Sod2-R | CTCGGTGGCGTTGAGATTGTT |
| Sod3-F | TTCTTGTTCTACGGCTTGCTACTG |
| Sod3-R | AGCTGGACTCCCCTGGATTT |
| Gapdh-F | AGGTCGGTGTGAACGGATTTG |
| Gapdh-R | GGGGTCGTTGATGGCAACA |
